# Hydroxychloroquine, a less toxic derivative of chloroquine, is effective in inhibiting SARS-CoV-2 infection in vitro

**DOI:** 10.1038/s41421-020-0156-0

**Published:** 2020-03-18

**Authors:** Jia Liu, Ruiyuan Cao, Mingyue Xu, Xi Wang, Huanyu Zhang, Hengrui Hu, Yufeng Li, Zhihong Hu, Wu Zhong, Manli Wang

**Affiliations:** 1 State Key Laboratory of Virology, Wuhan Institute of Virology, Center for Biosafety Mega-Science, Chinese Academy of Sciences, 430071 Wuhan, China; 2 National Engineering Research Center for the Emergency Drug, Beijing Institute of Pharmacology and Toxicology, 100850 Beijing, China; 3 University of the Chinese Academy of Sciences, 100049 Beijing, China

**Keywords:** Autophagy, Transcription

Dear Editor,

The outbreak of coronavirus disease 2019 (COVID-19) caused by the severe acute respiratory syndrome coronavirus 2 (SARS-CoV-2/2019-nCoV) poses a serious threat to global public health and local economies. As of March 3, 2020, over 80,000 cases have been confirmed in China, including 2946 deaths as well as over 10,566 confirmed cases in 72 other countries. Such huge numbers of infected and dead people call for an urgent demand of effective, available, and affordable drugs to control and diminish the epidemic.

We have recently reported that two drugs, remdesivir (GS-5734) and chloroquine (CQ) phosphate, efficiently inhibited SARS-CoV-2 infection in vitro^1^. Remdesivir is a nucleoside analog prodrug developed by Gilead Sciences (USA). A recent case report showed that treatment with remdesivir improved the clinical condition of the first patient infected by SARS-CoV-2 in the United States^2^, and a phase III clinical trial of remdesivir against SARS-CoV-2 was launched in Wuhan on February 4, 2020. However, as an experimental drug, remdesivir is not expected to be largely available for treating a very large number of patients in a timely manner. Therefore, of the two potential drugs, CQ appears to be the drug of choice for large-scale use due to its availability, proven safety record, and a relatively low cost. In light of the preliminary clinical data, CQ has been added to the list of trial drugs in the Guidelines for the Diagnosis and Treatment of COVID-19 (sixth edition) published by National Health Commission of the People’s Republic of China.

CQ (N4-(7-Chloro-4-quinolinyl)-N1,N1-diethyl-1,4-pentanediamine) has long been used to treat malaria and amebiasis. However, *Plasmodium falciparum* developed widespread resistance to it, and with the development of new antimalarials, it has become a choice for the prophylaxis of malaria. In addition, an overdose of CQ can cause acute poisoning and death^3^. In the past years, due to infrequent utilization of CQ in clinical practice, its production and market supply was greatly reduced, at least in China. Hydroxychloroquine (HCQ) sulfate, a derivative of CQ, was first synthesized in 1946 by introducing a hydroxyl group into CQ and was demonstrated to be much less (~40%) toxic than CQ in animals^4^. More importantly, HCQ is still widely available to treat autoimmune diseases, such as systemic lupus erythematosus and rheumatoid arthritis. Since CQ and HCQ share similar chemical structures and mechanisms of acting as a weak base and immunomodulator, it is easy to conjure up the idea that HCQ may be a potent candidate to treat infection by SARS-CoV-2. Actually, as of February 23, 2020, seven clinical trial registries were found in Chinese Clinical Trial Registry (http://www.chictr.org.cn) for using HCQ to treat COVID-19. Whether HCQ is as efficacious as CQ in treating SARS-CoV-2 infection still lacks the experimental evidence.

To this end, we evaluated the antiviral effect of HCQ against SARS-CoV-2 infection in comparison to CQ in vitro. First, the cytotoxicity of HCQ and CQ in African green monkey kidney VeroE6 cells (ATCC-1586) was measured by standard CCK8 assay, and the result showed that the 50% cytotoxic concentration (CC_50_) values of CQ and HCQ were 273.20 and 249.50 μM, respectively, which are not significantly different from each other (Fig. 1a). To better compare the antiviral activity of CQ versus HCQ, the dose–response curves of the two compounds against SARS-CoV-2 were determined at four different multiplicities of infection (MOIs) by quantification of viral RNA copy numbers in the cell supernatant at 48 h post infection (p.i.). The data summarized in Fig. 1a and Supplementary Table S1 show that, at all MOIs (0.01, 0.02, 0.2, and 0.8), the 50% maximal effective concentration (EC_50_) for CQ (2.71, 3.81, 7.14, and 7.36 μM) was lower than that of HCQ (4.51, 4.06, 17.31, and 12.96 μM). The differences in EC_50_ values were statistically significant at an MOI of 0.01 (*P* < 0.05) and MOI of 0.2 (*P* < 0.001) (Supplementary Table S1). It is worth noting that the EC_50_ values of CQ seemed to be a little higher than that in our previous report (1.13 μM at an MOI of 0.05)^1^, which is likely due to the adaptation of the virus in cell culture that significantly increased viral infectivity upon continuous passaging. Consequently, the selectivity index (SI = CC_50_/EC_50_) of CQ (100.81, 71.71, 38.26, and 37.12) was higher than that of HCQ (55.32, 61.45, 14.41, 19.25) at MOIs of 0.01, 0.02, 0.2, and 0.8, respectively. These results were corroborated by immunofluorescence microscopy as evidenced by different expression levels of virus nucleoprotein (NP) at the indicated drug concentrations at 48 h p.i. (Supplementary Fig. S1). Taken together, the data suggest that the anti-SARS-CoV-2 activity of HCQ seems to be less potent compared to CQ, at least at certain MOIs.

**Fig. 1:**
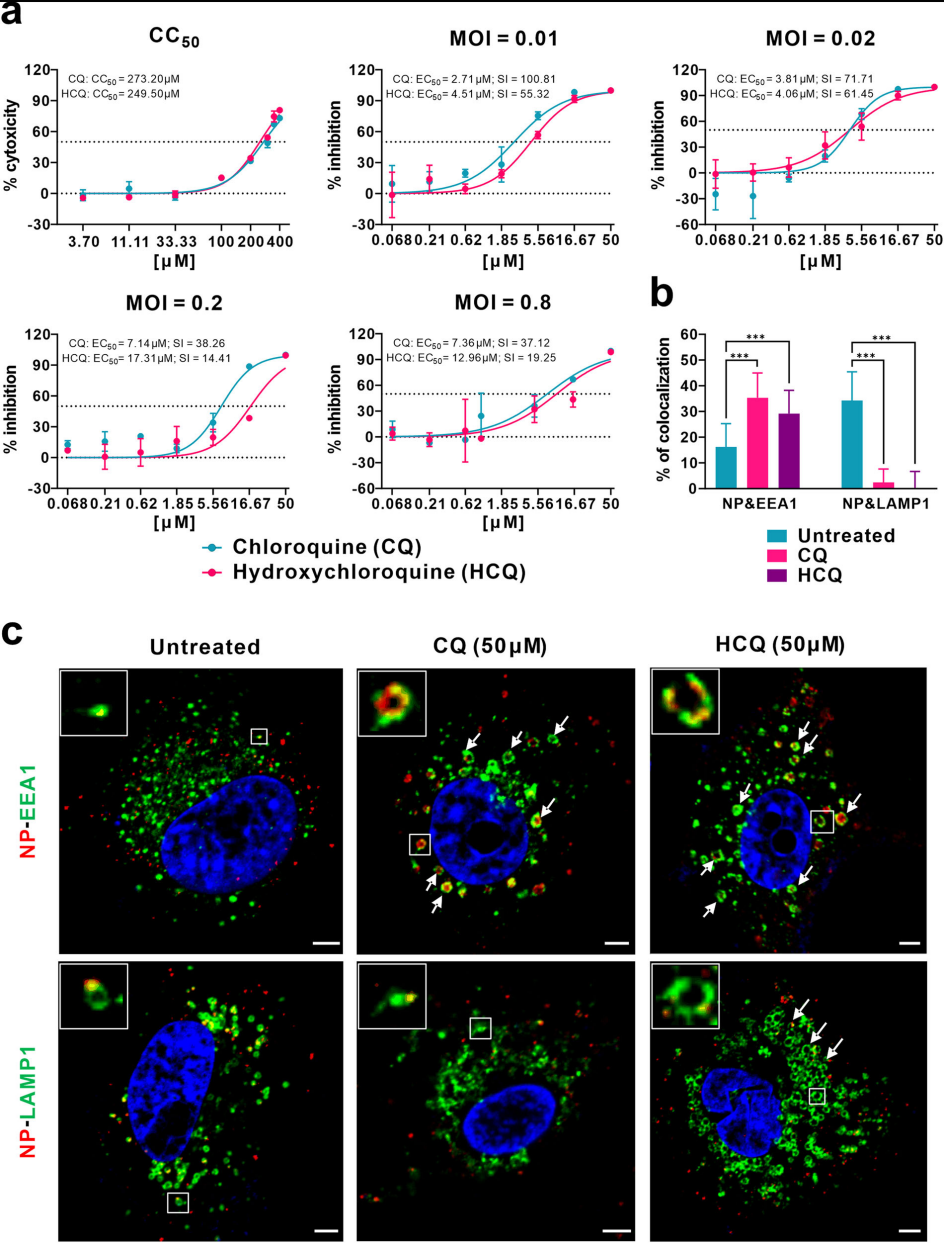
Comparative antiviral efficacy and mechanism of action of CQ and HCQ against SARS-CoV-2 infection in vitro. **a** Cytotoxicity and antiviral activities of CQ and HCQ. The cytotoxicity of the two drugs in Vero E6 cells was determined by CCK-8 assays. Vero E6 cells were treated with different doses of either compound or with PBS in the controls for 1 h and then infected with SARS-CoV-2 at MOIs of 0.01, 0.02, 0.2, and 0.8. The virus yield in the cell supernatant was quantified by qRT-PCR at 48 h p.i. *Y*-axis represents the mean of percent inhibition normalized to the PBS group. The experiments were repeated twice. **b**, **c** Mechanism of CQ and HCQ in inhibiting virus entry. Vero E6 cells were treated with CQ or HCQ (50 μM) for 1 h, followed by virus binding (MOI = 10) at 4 °C for 1 h. Then the unbound virions were removed, and the cells were further supplemented with fresh drug-containing medium at 37 °C for 90 min before being fixed and stained with IFA using anti-NP antibody for virions (red) and antibodies against EEA1 for EEs (green) or LAMP1 for ELs (green). The nuclei (blue) were stained with Hoechst dye. The portion of virions that co-localized with EEs or ELs in each group (*n* > 30 cells) was quantified and is shown in **b**. Representative confocal microscopic images of viral particles (red), EEA1^+^ EEs (green), or LAMP1^+^ ELs (green) in each group are displayed in **c**. The enlarged images in the boxes indicate a single vesicle-containing virion. The arrows indicated the abnormally enlarged vesicles. Bars, 5 μm. Statistical analysis was performed using a one-way analysis of variance (ANOVA) with GraphPad Prism (*F* = 102.8, df = 5,182, ***P < 0.001).

Both CQ and HCQ are weak bases that are known to elevate the pH of acidic intracellular organelles, such as endosomes/lysosomes, essential for membrane fusion^5^. In addition, CQ could inhibit SARS-CoV entry through changing the glycosylation of ACE2 receptor and spike protein^6^. Time-of-addition experiment confirmed that HCQ effectively inhibited the entry step, as well as the post-entry stages of SARS-CoV-2, which was also found upon CQ treatment (Supplementary Fig. S2). To further explore the detailed mechanism of action of CQ and HCQ in inhibiting virus entry, co-localization of virions with early endosomes (EEs) or endolysosomes (ELs) was analyzed by immunofluorescence analysis (IFA) and confocal microscopy. Quantification analysis showed that, at 90 min p.i. in untreated cells, 16.2% of internalized virions (anti-NP, red) were observed in early endosome antigen 1 (EEA1)-positive EEs (green), while more virions (34.3%) were transported into the late endosomal–lysosomal protein LAMP1^+^ ELs (green) (*n* > 30 cells for each group). By contrast, in the presence of CQ or HCQ, significantly more virions (35.3% for CQ and 29.2% for HCQ; *P* < 0.001) were detected in the EEs, while only very few virions (2.4% for CQ and 0.03% for HCQ; *P* < 0.001) were found to be co-localized with LAMP1^+^ ELs (*n* > 30 cells) (Fig. 1b, c). This suggested that both CQ and HCQ blocked the transport of SARS-CoV-2 from EEs to ELs, which appears to be a requirement to release the viral genome as in the case of SARS-CoV^7^.

Interestingly, we found that CQ and HCQ treatment caused noticeable changes in the number and size/morphology of EEs and ELs (Fig. 1c). In the untreated cells, most EEs were much smaller than ELs (Fig. 1c). In CQ- and HCQ-treated cells, abnormally enlarged EE vesicles were observed (Fig. 1c, arrows in the upper panels), many of which are even larger than ELs in the untreated cells. This is in agreement with previous report that treatment with CQ induced the formation of expanded cytoplasmic vesicles^8^. Within the EE vesicles, virions (red) were localized around the membrane (green) of the vesicle. CQ treatment did not cause obvious changes in the number and size of ELs; however, the regular vesicle structure seemed to be disrupted, at least partially. By contrast, in HCQ-treated cells, the size and number of ELs increased significantly (Fig. 1c, arrows in the lower panels).

Since acidification is crucial for endosome maturation and function, we surmise that endosome maturation might be blocked at intermediate stages of endocytosis, resulting in failure of further transport of virions to the ultimate releasing site. CQ was reported to elevate the pH of lysosome from about 4.5 to 6.5 at 100 μM^9^. To our knowledge, there is a lack of studies on the impact of HCQ on the morphology and pH values of endosomes/lysosomes. Our observations suggested that the mode of actions of CQ and HCQ appear to be distinct in certain aspects.

It has been reported that oral absorption of CQ and HCQ in humans is very efficient. In animals, both drugs share similar tissue distribution patterns, with high concentrations in the liver, spleen, kidney, and lung reaching levels of 200–700 times higher than those in the plasma^10^. It was reported that safe dosage (6–6.5 mg/kg per day) of HCQ sulfate could generate serum levels of 1.4–1.5 μM in humans^11^. Therefore, with a safe dosage, HCQ concentration in the above tissues is likely to be achieved to inhibit SARS-CoV-2 infection.

Clinical investigation found that high concentration of cytokines were detected in the plasma of critically ill patients infected with SARS-CoV-2, suggesting that cytokine storm was associated with disease severity^12^. Other than its direct antiviral activity, HCQ is a safe and successful anti-inflammatory agent that has been used extensively in autoimmune diseases and can significantly decrease the production of cytokines and, in particular, pro-inflammatory factors. Therefore, in COVID-19 patients, HCQ may also contribute to attenuating the inflammatory response. In conclusion, our results show that HCQ can efficiently inhibit SARS-CoV-2 infection in vitro. In combination with its anti-inflammatory function, we predict that the drug has a good potential to combat the disease. This possibility awaits confirmation by clinical trials. We need to point out, although HCQ is less toxic than CQ, prolonged and overdose usage can still cause poisoning. And the relatively low SI of HCQ requires careful designing and conducting of clinical trials to achieve efficient and safe control of the SARS-CoV-2 infection.

## Supplementary information

Supplemental Materials

## Supplementary information

**Supplementary Information** accompanies the paper at (10.1038/s41421-020-0156-0).
