# Supplementary material for: Hydroxychloroquine, a less toxic derivative of chloroquine, is effective in inhibiting SARS-CoV-2 infection in vitro
Source: Cell Discov. 2020 Mar 18;6:16. doi: 10.1038/s41421-020-0156-0 (PMC7078228; doi:10.1038/s41421-020-0156-0)
Supplement: Supplementary file 1 — Supplemental Materials [file 41421_2020_156_MOESM1_ESM.pdf]

Supplementary information

Hydroxychloroquine, a less toxic derivative of chloroquine, is effective in inhibiting SARS-CoV-2 infection *in vitro*

Jia Liu[1#], Ruiyuan Cao[2#], Mingyue Xu[1, 3#], Xi Wang[1], Huanyu Zhang[1, 3], Hengrui Hu[1, 3], Yufeng Li[1, 3], Zhihong Hu[1*], Wu Zhong[2*], Manli Wang[1*]

[1]State Key Laboratory of Virology, Wuhan Institute of Virology, Center for Biosafety Mega-Science, Chinese Academy of Sciences, Wuhan 430071, P. R. China; [2]National Engineering Research Center for the Emergency Drug, Beijing Institute of Pharmacology and Toxicology, Beijing 100850, China; [3]University of the Chinese Academy of Sciences, Beijing 100049, P. R. China.

#These authors contributed equally:

Jia Liu[1#], Ruiyuan Cao[2#], Mingyue Xu[1#]

*Correspondence:

Prof., Dr. Manli Wang; E-mail: wangml@wh.iov.cn

Prof., Dr. Wu Zhong; E-mail: zhongwu@bmi.ac.cn

Prof., Dr. Zhihong Hu; E-mail: huzh@wh.iov.cn

Materials and Methods

Cells and virus

African green monkey kidney Vero E6 cell line (ATCC, no. 1586) was cultured in minimum Eagle's medium (MEM; Gibco/Invitrogen) supplemented with 10% fetal bovine serum (FBS; Gibco/Invitrogen) at 37°C with 5% CO_2 atmosphere. A clinical strain of severe acute respiratory syndrome coronavirus 2 (SARS-CoV-2) (nCoV-2019BetaCoV/Wuhan/WIV04/2019)[1] was propagated in Vero E6 cells, and virus stock was maintained at -80°C. All the infection experiments were performed in a biosafety level-3 (BLS-3) laboratory.

Virus titer was determined by a standard tissue culture infectious dose ($TCID_{50}$) endpoint dilution assay using immunofluorescence staining method with anti-nucleocapsid protein (NP) rabbit serum (1:1000 dilution) and Alexa 488-labeled goat anti-rabbit IgG (1:500 dilution; Abcam) as the primary and the secondary antibody, respectively.[1]

Evaluation of antiviral activities of the drugs

To analyze the antiviral activities of chloroquine phosphate (CQ, Sigma-Aldrich, no. C6628) and hydroxychloroquine sulfate (HCQ, MCE, no. HY-B1370), the cytotoxicity of the two drugs on Vero E6 cells was first determined using cell counting kit-8 (CCK8) (Beyotime, China) according to manufacturer's protocol. Then, Vero E6 cells (1×10^5 cells/well) cultured in 48-well cell-culture plates were

pre-treated with the different concentrations of the two compounds for 1 h at 37 °C, followed by infection with virus at different MOIs (0.01, 0.02, 0.2, and 0.8) for 2 h. Subsequently, the virus-drug mixture was removed and cells were extensively washed with PBS. Then, the fresh drug-containing medium was added and further maintained until 48 h p.i., and the virus yield in the infected cell supernatant was quantified by quantitative real-time RT-PCR (qRT-PCR) (Takara, Cat no. 9766) as described previously.[2] Briefly, total viral RNA was isolated and cDNA was synthesized by reverse transcription. Quantitative PCR was performed using cDNA as the template with specific primers against the receptor binding domain (RBD) of viral spike gene. A standard curve was generated by using serially-diluted plasmid standards. After determining viral RNA copies in each group by qRT-PCR, the dose-response curves were plotted by using GraphPad Prism 6 software.

Time-of-addition experiment

Time-of-addition experiment of CQ and HCQ were performed as previously. [2] Briefly, Vero E6 cells (1×10^5 cells/well) were treated with CQ (10 μM), HCQ (30 μM) or PBS control at different stages of virus infection (Entry, Full-time, and Post-entry), and then infected with virus at an MOI of 0.07. At 16 h p.i., virus yield in the infected cell supernatant was quantified by qRT-PCR. The expression of viral NP in the infected cells was analyzed by immunofluorescence analysis (IFA) using anti-NP rabbit sera (1:1000 dilution) and Alexa 488-labeled goat anti-rabbit IgG (1:500 dilution; Abcam) as the primary and the secondary antibody, respectively. The nuclei

were stained with Hoechst 33258 dye (Beyotime, China). The cells were imaged by fluorescence microscopy.

The mechanism of CQ and HCQ in inhibition of virus entry

To analyze the mode of action of CQ and HCQ in inhibiting virus entry, co-localization analysis of virions and early endosomes (EEs) or endolysosomes (ELs) were carried out. Vero E6 cells (2.5×10^5 cells/well) cultured in 35-mm glass-bottom culture dishes were pre-treated with CQ (50 μM), HCQ (50 μM), or PBS control for 1 h before virus attachment, and then incubated with SARS-CoV-2 (MOI = 10) at 4 °C to allow virus binding for 1 h. After being washed three times with pre-chilled PBS, the cells were further cultured with fresh drug-containing medium at 37 °C for 90 min, and then fixed and subjected to IFA.

For co-localization analysis of virions and $EEA1^+$ EEs, the fixed cells were incubated with rabbit sera against NP (anti-NP, 1:500 dilution) and mouse anti-EEA1 antibody (1:100 dilution, Cell Signaling Technology) as the primary antibodies, and then stained with Alexa 647-labeled goat anti-rabbit IgG (1:500 dilution; Abcam) and Alexa 488-labeled rabbit anti-mouse IgG (1:500 dilution; Abcam) as the secondary antibodies, respectively. For co-localization analysis of virions and $LAMP1^+$ ELs, mouse monoclonal antibody against NP (1:100 dilution) and rabbit anti-LAMP1 antibody (1:500 dilution, Cell Signaling Technology) were used as the primary antibodies, and Alexa 647-labeled rabbit anti-mouse IgG (1:500 dilution; Abcam) and

Alexa 488-labeled goat anti-rabbit IgG (1:500 dilution; Abcam) were used as the secondary antibodies, respectively. Fluorescence images were obtained by using a confocal microscope (Nikon A1RMP two-photon microscope). Then, the proportions of SARS-CoV-2 particles (yellow) co-localized with EEs or ELs (green) to all particles (red) in the cells were quantified and analyzed (n > 30) by image J (Colocation Threshold plugin).

References:

1. Zhou, P. et al. A pneumonia outbreak associated with a new coronavirus of probable bat origin *Nature*. doi: 10.1038/s41586-020-2012-7 (2020).
2. Wang, M. et al. Remdesivir and chloroquine effectively inhibit the recently emerged novel coronavirus (2019-nCoV) in vitro. *Cell Res.* doi:10.1038/s41422-020-0282-0 (2020).

Table S1. The EC_{50} values with 95% confidential intervals (95% CI) of CQ and HCQ at different MOIs.

MOI	CQ	HCQ	*P* value
	EC_{50} (95% CI)		
**0.01**	2.71 (1.94 ~ 3.67)	4.51 (3.48 ~ 5.80)	< 0.05
**0.02**	3.81 (2.54 ~ 5.62)	4.06 (2.98 ~ 5.51)	> 0.05
**0.2**	7.14 (5.29 ~ 9.31)	17.31 (12.01 ~ 23.23)	< 0.001
**0.8**	7.36 (4.70 ~ 11.23)	12.96 (7.82 ~ 20.56)	> 0.05

The data were fitted to the nonlinear regression with selected unshared parameter parameters. The EC_{50} values were compared with Extra sum-of-squares *F* test.

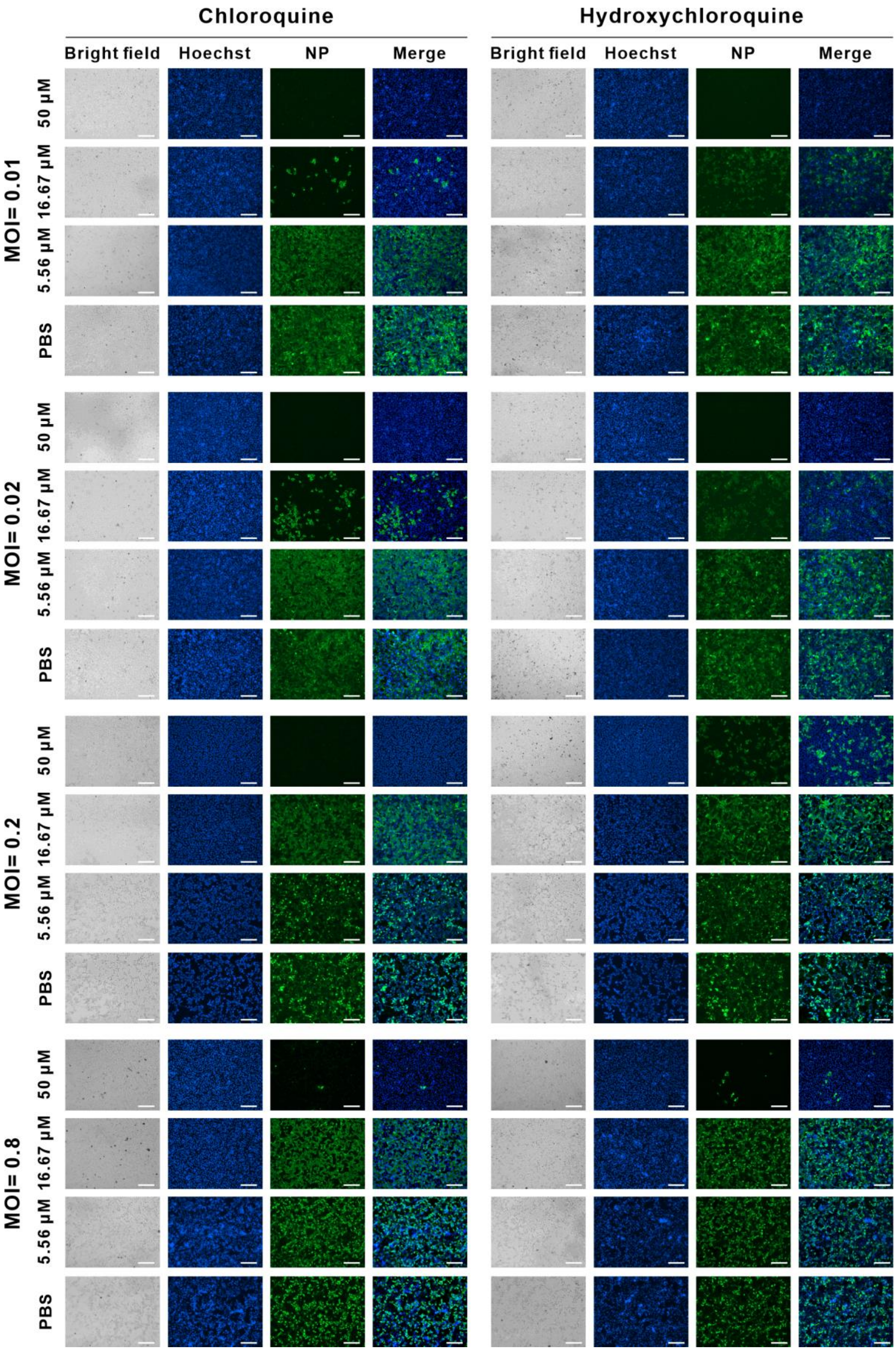

Fig. S1. Immunofluorescence microscopy of virus infection upon treatment of CQ and HCQ. Vero E6 cells untreated or pretreated with different doses of the two compounds for 1h, and infected with SARS-CoV-2 at MOIs of 0.01, 0.02, 0.2, and 0.8, respectively. At 48 h p.i., the infected cells were fixed, and the expression of NP (green) were detected by IFA with anti-NP rabbit sera and Alexa 488-labeled goat anti-rabbit IgG as the primary and the secondary antibody, respectively. The nuclei (blue) were stained with Hoechst dye. Bars, 200 μm.

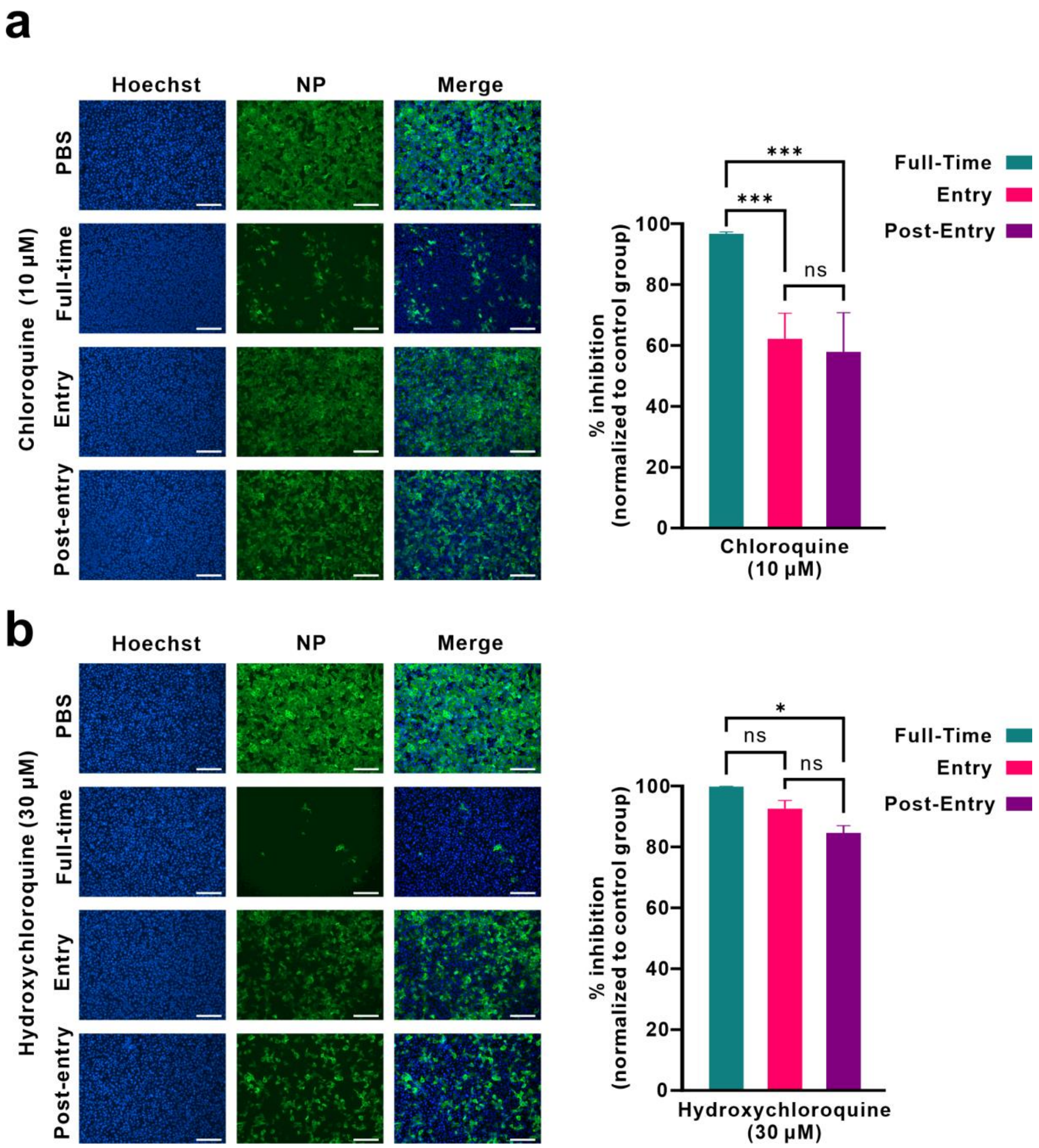

Fig. S2. Time-of-addition experiment of CQ (a) and HCQ (b). For "Entry" treatment, Vero E6 cells were pretreated with the drugs at the indicating concentrations for 1 h prior to viral attachment, followed by being infected with virus at an MOI of 0.07 for 2 h. Then, the supernatant was replaced with fresh culture medium without drugs and maintained till the end of the experiment. For "Full-time" treatment, drug treatment and virus infection were performed as the "Entry" treatment. After removal of the supernatant, fresh drug-containing medium was added to the cells until the end of the experiment. For "Post-entry" experiment, virus was added to the cells in the absence of drugs to allow infection for 2 h, and then replaced with drug-containing medium and maintained until the end of the experiment. At 16 h p.i., the expression of NP (green) in the infected cells was analyzed by IFA (the left panels) and virus yield in the infected cell supernatant was quantified by qRT-PCR (the right panels). The nuclei (blue) were stained with Hoechst dye. Bars, 200 μm. Statistical analysis was performed using a two-way analysis of variance (ANOVA) with GraphPad Prism.***, $P < 0.001$; *, $P < 0.05$; ns, not significant.
